# Editorial: Translational research in pediatric respiratory diseases: From bench to bedside

**DOI:** 10.3389/fped.2022.1114549

**Published:** 2023-01-06

**Authors:** Diego R. Hijano, Damián Alvarez-Paggi, Mauricio T. Caballero

**Affiliations:** ^1^ Department of Infectious Diseases, St. Jude Children’s Research Hospital, Memphis, TN, United States; ^2^ Consejo Nacional de Investigaciones Científicas y Técnicas (CONICET), Buenos Aires, Argentina; ^3^ Fundación INFANT, Buenos Aires, Argentina

**Keywords:** bronchiolitis, respiratory syncytal virus, ashtma, cystic fibrosis, sinusitis, CLCA1, translational research, respiratory diseases

**Editorial on the Research Topic** Translational research in pediatric respiratory diseases: From bench to bedside

Acute and chronic respiratory diseases account for the majority of the morbidity and mortality burden in children worldwide (1). Translational research can provide a more accurate understanding of pathophysiological mechanisms of illnesses, allowing to design new preventative, diagnosis and treatment strategies faster than by basic or clinical approaches alone (2, 3). Several respiratory diseases have clinical presentations that are far from uniform (4). For instance, bronchiolitis presents with overlapping symptoms of varying intensity and these variations can be observed with more than a dozen of viruses with different clinical phenotypes (4). Similarly, the syndrome we refer to as asthma can be a consequence of a wide range of different underlying mechanisms of disease (3). For that reason, we developed a translational research topic to bring together influential researchers in the field of pulmonary diseases in pediatrics encompassing a bench-to-bedside approach. The collection of articles included in this special issue reflect our vision on how to advance the field of respiratory health in children.

The role of toll-like receptor 4 (TLR4) D299G single nucleotide polymorphism (snp) in the severity of respiratory syncytial virus (RSV) has been well described during recent years (5). This TLR4 snp generates an amino acid substitution in the ectodomain associated with an alteration of the responses to different stimuli (5). Low exposure to endotoxins in infants with D299G snp presents a 9-fold greater risk of hospitalization due to RSV infection vs. the rest of the term population (6). *Ascaris lumbricoides* infection is common in tropical countries and it has been associated with asthma and recurrent wheezing (7, 8). On the grounds of this, Buendía et al. have explored in this topic supplement the association between this TLR4 snp in infants with *Ascaris lumbricoides* infection and RSV-associated disease severity (9). Interestingly, the authors have found that although positive anti-Ascaris IgE levels in serum were significantly associated with the probability of developing severe bronchiolitis, the presence of anti-Ascaris antibodies in the subgroup of infants with TLR4 D299G snp was significantly associated with associated with lower risk of severe bronchiolitis due to RSV (9). This finding adds supportive data for future characterization of infants with TLR4 snp. Furthermore, the detection of TLR4 D299G snp might be a relevant screening tool to assess risk for severe disease, as well as interventions under development against RSV disease.

Lopez et al. used clinical, virologic and immunologic data to diagnose bacterial sinusitis by predicting the presence of bacteria in the nasopharynx (10). They enrolled 174 children between 2 and 12 years of age with clinical diagnosis of sinusitis. Upper respiratory tract infections are often due to viral infection and are the most common causes of antibiotic misuse (11, 12) Tools that can better define patients who may benefit from antibiotics from those who will not, have the potential to significantly impact clinical care and aid in the fight against the threat of antimicrobial resistance (13). While authors did not find significant differences among patients with and without bacterial sinusitis, they showed that point of care testing from a single swab can be used to characterize the local microbiologic and immunologic profile of children with acute illness opening the potential for other tests that may in fact be critical in guiding antibiotic use.

Cystic fibrosis (CF) is a progressive chronic disease caused by recessive genetic variants in the cystic fibrosis transmembrane conductance regulator (CFTR) gene that affects lungs, pancreas, and other organs (14). It is the most common life-threatening inherited disorder of children that affects 1 in 3,300 live births in the world (15). Despite CF being a serious disease that can be associated with early mortality, the epidemiology and disease prognosis have changed in the last 20 years (14). In fact, the survival of CF patients has greatly improved in recent decades. In this topic Castro e Garcia et al. have provided a summary of improvements on diagnostics and therapeutics (16). In this review authors described how basic science research has generated improvements in CF prognosis. Additionally, authors have mentioned the impact of cystic fibrosis transmembrane conductance regulator (CFTR) protein modulators on life expectancy for almost all individuals with CF.

CLCA1 is a member of the CLCA (calcium-activated chloride channel regulator) family, and plays a key role in the pathogenesis of mucus hypersecretory-associated respiratory diseases, such as asthma (17). CLCA1 modulates mucus dynamics and homeostasis in multiple environments, such as intestinal and respiratory epithelia. In this topic, Xu et al. (18) investigated associations between CLCA1 expression and IL-13 levels, which is the main biomarker of airway and blood eosinophilia, a main characteristic of type 2 asthma (19). To this end, the authors designed a full “bench-to-bedside” experimental approach. They showed that CLCA1 expression in epithelial tissue from asthmatic children was significantly increased when compared to *tissue from non-asthmatic* individuals. In addition, asthmatic individuals had increased IL-13 and IL-4 levels. A correlation between CLCA1 and IL-13 levels was then explored *in vitro* showing that IL-13 stimulation upregulates expression of CLCA1. Finally, the potential mechanism of such interactions was explored by siRNA-mediated knockdown of CLCA1, which resulted in attenuation of IL-13 induced bronchial epithelial cell activity and reduction of cell apoptosis. These results strongly suggest that CLCA1-mediated IL-13 may be a key driver in pediatric asthma, paving the way to identify novel potential therapeutic targets in this population.

Manti and Piedimonte provide a comprehensive and up-to-date view of a critical association that has come to prominence during the last decades: the link between RSV infection and asthma (20). Although severe RSV infection has been associated with long term complications such as impaired lung function, recurring wheezing and asthma, whether RSV lower respiratory tract infection causally affects the possibility of developing asthma during childhood has not been determined, and the possible mechanisms are poorly characterized (21). In their review article, the authors help organize and bring clarity to this matter by providing a full description of RSV structure including the key players in the host-pathogen interaction, at the cellular, tissular and immunological level. Interestingly, the often-neglected effect of RSV infection on neurological pathways is covered extensively, highlighting multiple cellular mechanisms that impact directly on the dysregulation of the neural control of airway smooth cells and the link with asthma onset. Finally, a thorough description of *in vivo* and human models for RSV and non-allergic asthma is provided. This section should be of reference for anyone interested in setting up a translational experiment exploring the role of RSV infection and development of asthma, as multiple results obtained from both models are provided, including a critical discussion of the advantages and shortcomings inherent to each specific approach. Finally the authors support a “two-hit” model for the development of RSV-induced asthma involving the co-existence of at least two factors from the trio of individual, developmental, and environmental variables, trying to shed light on the usually contradicting results that look for linear associations between different factors (22).
